# The Life Mission Theory V. Theory of the Anti-Self (the Shadow) or the Evil Side of Man

**DOI:** 10.1100/tsw.2003.117

**Published:** 2003-12-11

**Authors:** Soren Ventegodt, Niels Jorgen Andersen, Joav Merrick

**Affiliations:** The Quality of Life Research Center, Copenhagen K, Denmark

**Keywords:** quality of life, QOL, philosophy, purpose of life, shadow, evil, higher self, suicide, enlightenment, religion, human development, holistic medicine, public health, Denmark

## Abstract

According to the life mission theory, the essence of man is his purpose of life, which comes into existence at conception. This first purpose is always positive and in support of life. This is not in accordance with the everyday experience that man also engages in evil enterprises born out of destructive intentions. This paper presents a theory about the evil side of man, called the “anti-self” (the shadow), because it mirrors the self and its purpose of life. The core of the anti-self is an evil and destructive intention opposite to the intention behind the life mission. The evil side of man arises when, as the life mission theory proclaims, man is denying his good, basic intention to avoid existential pain. The present theory of the anti-self claims that all the negative decisions accumulated throughout the personal history, sum up to a negative or dark anti-self, as complex, multifaceted, and complete as the self. All the negative decisions taken through personal history build this solid, negative, existential structure. The anti-self, or shadow as Carl Gustav Jung used to call it, is a precise reflection of man's basically good and constructive nature. When mapped, it seems that for most or even for all the many fine talents of man, there is a corresponding evil intention and talent in the person's anti-self. As man is as evil as he is good, he can only realize his good nature and constructive talents by making ethical choices. Ethics therefore seem to be of major importance to every patient or person engaged in the noble project of personal growth. Understanding the nature and structure of the evil side of man seems mandatory to every physician or therapist offering existential therapy to his patient. The theory of anti-self makes it possible to treat patients with destructive behavioral patterns, who want to be good deep in their heart, by helping them let go of their evil intentions. The anti-self also seems to explain the enigma of why human beings often commit suicide. Integrating the shadow often leads to dramatic, subjective experiences of ubiquitous light in an “unpersonal” form, of enlightenment, or of meeting light and consciousness in a personal, universal form, known as G-d.

## INTRODUCTION

Man has a free will, acknowledged by philosophers of all times, and by using this will man can either do good or become engaged in evil intentions and by doing so, assumes often grotesque and inhuman forms. Numerous are the examples of such demonic beings, like Lord Dracula, Hannibal the Cannibal, or Jack the Ripper. What seems to be even scarier is that we daily face seemingly normal men and woman that are caught as child molesters, criminals of war, rapists, etc. in the media. Everybody seems to have the potential to be evil, and it seems as easy to be evil as it is to be good; hence the existential choice and the free will.

History is packed with examples of people abusing their power to live out their dark side. During the Inquisition, it is estimated that between five and ten million innocent people were burned alive as witches in the name of Jesus by the ministers of Europe[1,2] and six million Jews (including one million children) were killed by the Germans during the Holocaust. Thus, even religious ministers, who should be the representatives of G-d, the most devoted guards of the good, cannot reproach themselves from the shadow, the dark side of man, or the evil side.

The yoke of heaven, or the abstract guiding principle of mankind, must be for everyone to know what his task is in this world and to understand the principles of the universe, or the ways of G-d, the Divine ideas, and the way mankind should choose in order to achieve his purpose in life[3,4,5,6]. It is said:

There has been a lot of research in the nature and source of the evil motives of man. Sigmund Freud (1856–1939) explained the evil side of man as a natural force: a basic urge or instinct of moving towards death. Along with the sexual instinct, this constitutes the two essential urges in man, the only two real motivators[4]. Carl Gustav Jung (1875–1961), the grand student of Freud, studied the shadow intensely and described it perhaps better than anyone[5]. Jung had a complicated relationship with the shadow, since he apparently — on the one hand — thought that the shadow, the dark side of man, contains a substantial developing potential that is set free when we attempt to integrate the shadow, but — on the other hand — he believed that the shadow can never be completely eliminated or defeated.

Studies of the dynamics of therapeutic interventions with existential therapy based on the life mission theory[6,7,8,9] show that the dark side of man has a relatively simple structure. When the structure of the shadow is worked out and eventually mapped during therapy, it can be integrated, if the patient chooses to let go of it[8]. The understanding of the general nature and structure of the dark side of man is important in this work with the patient.

If a person succeeds to re-intend his true life mission, he will often be almost ecstatically happy in his sensation of having found himself and his inner truth. Soon after he will often feel pain, because he reaches contact with the original situation, where he was unable to make a difference. The strong intensity of the positive emotions that is found around the purpose of life can be explained in terms of all the good that is repressed in man. The often overwhelming intensity of the life pain explains why the mission of life is often repressed throughout life and why the evil side is often preferred instead of the good.

## SELF AND ANTI-SELF, LIFE PURPOSE AND ANTI-PURPOSE

According to the life mission theory[6], the life purpose is so painful in the beginning of our life that we end up denying and repressing it. We repress it by intending the opposite of the original positive intention behind our purpose of life. This very negative intention is also repressed as we assume another constructive purpose of life. Soon after, this new positive purpose is also denied by a negative intention and so forth. So during our upbringing, both the positive and the negative intentions of man are repressed from the surface of consciousness, and forced into the famous, but mysterious unconsciousness.

When carefully sought, the repressed intentions can be found as "gestalts" carrying both cognitive and emotional data, in split up parts or "pockets" of our biological existence. In body therapy these pockets are known as "blockages", tissue areas with a strange tense quality to them. The blockages release the gestalts to the consciousness of the person when competently contacted in the therapy. It seems that these gestalts, even after many years, are still potentially very active, and they surface as soon as the person calls on the destructive intentions, as it can happen in a moment of fury or anger. The dark side then takes over and the person is out of conscious control for a period of time. He or she seems to be very present and awake, but is not really, as the gestalts have taken the person and drawn him back in time.

The philosophical question here is if the person could have acted otherwise with a higher ethical standard. This is a very difficult issue giving birth to the discussion of insanity in the moment of crime, which in many countries releases the person from responsibility according to the law.

Most normal persons of our time have most of their good as well as most of their evil intentions suppressed. All the repressed, positive intentions in a person are basically pulling the same way, and are summed up to our unconscious, good side. In the same way, all our repressed, negative intentions are summed up to our unconscious, evil side. In the normal person, who does not know himself very well, the good and the evil forces are mostly unconscious and of almost the same size, since they quite accurately equilibrate each other[10]. The unconscious man functions opportunistically, since he is shifting between the good and the evil intentions, from situation to situation, according to what is most suitable in the given situation in relation to survival and satisfaction of needs. Such a person does not take conscious ownership of his own intentions; therefore he projects both the good and the evil to the surrounding world and its people, and therefore he cannot live his life with much strength. He is not in control of his own existence[11].

When man becomes more conscious, he acknowledges that he has to choose between the good and the evil. As he grows, he must confront the basic ethical choice in life. Since the two sides, good and evil, are balancing each other, man is free to choose between the good and the evil; the choice decides which side will consciously be lived out. The side that is not chosen by the half-conscious man will not disappear, however, but is projected to the surrounding world. A person who chooses to be good is in this phase of his personal growth denying his own hidden evilness, and can now only indirectly observe this denied black side, which appears as evilness and darkness around him. Unfortunately the repressed evil intentions are still highly active in man and more so if he consciously chooses the good; as he unconsciously still balances the good and the evil to avoid the severe existential pain that according to the life mission theory is inevitably linked to the denied life purpose. Very often the "ethical person" ends up in a colorful and dramatic battle with his own shadow, unwillingly and unconsciously causing harm to self and others[12].

The conclusion is that evilness is difficult to get rid of. Even the person who consciously chooses to be good will often unconsciously be evil, but still this person is likely to be far less evil than the opportunist, who lives completely without ethics. The person who contrarily chooses the evil and denies the good will be unconsciously good in spite of this, but still worse than the opportunist. The hidden goodness and the internal battle between the good and the evil in man explains why the person, who in his madness chooses the evil path to solve his problems, is often not successful in living out his evilness, but ends up stuck in his own existential problems[13].

The person who admits that he contains positive as well as negative intentions in his unconsciousness, and strives to embrace both with his existence, can in time observe and acknowledge both sides in himself and can thereby gradually take responsibility for all aspects of his existence. This person develops and grows, and will gradually be able to let go of the negative decisions that sum up his dark side, his anti-self, or the shadow-side that prevents his ability to live out his mission in life and express his true self. Existential therapy, which makes use of this knowledge about self and anti-self, can therefore help people to integrate their dark, negative side, and express themselves fully[14].

## THE CREATION OF THE ANTI-SELF ("THE SHADOW")

In our true and natural state of being, all our intentions and talents are centered around our life mission. This set of intentions is how we express our true self, but very often only a fraction of our natural power and potential is lived as we live through our ego[15,16,17]. Our ego appears as we deny our purpose of life and our secondary purposes, one by one, with a row of negative decisions. This continues until only a small part of our inherent nature and talents is left operational.

Most people over 20 years of age actively deny all, or almost all, of the central aspects of their true self. This happens as a consequence of adaptation. This way everybody ends up having a white aspect of the self for every talent and good intention in life, and a corresponding black aspect for every evil intention and destructive talent. The evil outbalances the good. Man is at peace, but often bored with life, and many people of our time experience their existence as almost meaningless.

As man develops his consciousness about himself, he moves from being chaotic to polarized, as he obtains the ability to discriminate between the good and the evil within himself. Now he sees that all the negative, black intentions are basically turned towards life, while all the positive, white intentions are supporting life. Black and white are outbalancing each other, and the dark side has annulated the purpose of life.

In other words, our black, or self-destructive, side is appearing as our dark shadow, which precisely is cowering our positive self (the "life mission flower"[15], except the little place that is left back without denial and which gives rise to our ego [see Figs. 1 and 2 in 15]). The shadow is, accordingly, technically seen as the difference between our true self and our ego.

## THE ANATOMY OF THE SHADOW

The shadow, hence, is a set of destructive intentions, which is organized in exactly the same way as our true self, where the set of good intentions are assembled in the life mission flower around the purpose of life[15]. The center of shadow is the intention destroying the purpose of life most directly, called the anti-life–mission or anti-life–purpose. The shadow therefore appears as a negative copy of the personality, as its black parallel (see Fig. 1). The purpose of establishing the shadow during our personal history was to weaken the good intentions that could not be realized in early childhood and therefore became too painful.

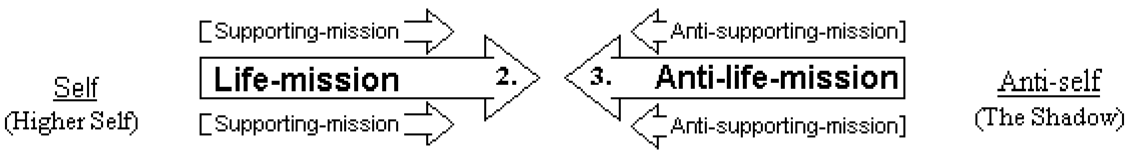

**FIGURE 1.** The life-mission, as well as all the supporting missions, is repressed, and man does not know himself. The repression has happened through negative anti-decisions, which outbalance the positive missions. The negative decisions are hence anti-self intentions that are organized around the most basic self-denial — the negative decisions that are here called anti-life–missions. All of these anti-self intentions exist in the parts of us that are still active, even though they are repressed; they sum up to be one self-destructive intentional structure in us, here called the anti-self.

Because the shadow has developed into a copy of the true self, it looks as if the whole shadow can be given life, if man — using the power of his free will — chooses to adopt the negative perspective, which lies in the denial of his life mission. The new mission of life for this person is the evil anti-life–mission. Now, in the worst-case scenario, we have the serial killer in action.

## AN EXAMPLE: FROM “I AM GOOD” TO “I AM EVIL”

An easy, understandable example is the following: This person had as a life mission the sentence “I am good”. The anti-life purpose was the complete opposite: “I am evil”. This person used one of the two in an opportunistic way. If he chose to be good he would repress his evil side. If he chose to be evil, he would repress his good side. The original purpose of this destructive intention was to outbalance the intention about being good, so that the pain of not being able to do any good was eliminated early in life.

## WHAT MAKES MAN CHOOSE EVIL?

There seem to be huge emotional advantages for the person to be evil, since this identity is often far less existentially painful, because you inflict the pain on somebody else. This is opposite to being good, where it is often you who suffers. If a person chooses consciously to be evil, all of the black shadow-flower will be this new person’s personality, to a high degree free of emotional pain[6].

A feeling of enormous power is set free in man when the anti-mission is re-intended and all of the repressed, negative supporting missions and destructive talents that are organized around the anti-mission are activated. This is typically experienced as a wild roar, even ecstasy, of raw power and animalistic strength[19]. Most people have a sense of reason, which makes them live in the head, preventing both the extremely constructive as well as the extremely destructive perspective of life to manifest itself.

The experience of intensity and energy without any life pain can peak in a sheer delight of domination; “Satanic pleasure” seems to be the most appropriate designation for this intense joy of the dark side.

## THE DYNAMIC OF THE SHADOW

All the good in man comes from his purpose of life, which expresses the essence of his true self. According to the life mission theory, the mission is always good and stable throughout life. It is often so repressed and suppressed in man that it cannot be known, expressed, or lived out. The evil exists, correspondingly, as an anti-mission that expresses the opposite of our profound self. The anti-self is as stable as the self, but has a destructive nature and intention.

The intentions of average people from the western world of today are not focused towards either the positive or the negative. Life is not guided either by the mission or the anti-mission. Rather the intention is presented as an in-between of these two. This means that the ordinary, unconscious person does not have much drive, energy, or enthusiasm. His direction in life is confused. Both the good and the evil are seen as non-self, and projected on the outside world. Thus man does not usually ascribe to himself any special strengths or talents, or any specific significance.

Many people of our time see themselves as having a great potential and work in order to express this potential through personal development. Re-intending the original mission of life and developing a more conscious relationship to existence and a clearer recognition of who one is, raises the experience of joy of life and the level of personal energy. But personal growth will also reawaken the original life pain.

A lot of people who search for themselves are therefore experiencing great difficulties. At some time, everybody seemingly has to face their dark side — their own shadow. Christianity tells us the story about how Jesus was tempted in the desert by the devil’s promises of immense power and sovereignty, but finally chose love and the good[20,21].

When it comes to patients that are working with themselves, but seriously ill, it is especially important for the physician to know the mechanisms that are prevailing in such a situation. Supporting the patient so that he can develop himself might lead to the patient entering the troublesome existential phase. The optimal approach for the patient is to take responsibility for his whole being including both the light and the shadow, and at the same time strive to express his white essence, the meaning and purpose of his life. To do so, he has to constantly reflect on his own participation in all aspects of reality. As he acknowledges that he contains both good as well as evil, he must carefully scrutinize all of his intentions to see if they are constructive or destructive.

The learning position is difficult because we are fragmented in a number of parts with almost their own life, until we have confronted and integrated our historical life pains. The fragmentation happens because of our contradictory consciousness.

Everyone owns a huge number of black and white intentions, and we all live the black as much as the white, even though we are not aware of it. The conscious, integrated, and transcendent position is a tough and challenging position that brings up as much historical life pain to the surface as we have the resources to handle. Without a supply of external resources such as holding[7], the development will be slow and painful. Interestingly enough, excess of resources means that a person can move quickly forward and integrate considerable parts of repressed life pain. Hereby he will let go of a lot of negative decisions, and the shadow will become gradually smaller.

Jung did not believe that the shadow could be integrated to a situation where it disappeared, but this is exactly the classical ideal of enlightenment[5,19,20,21]. From a theoretical point of view, all life pains can be integrated until the anti-mission is gone and the fragments that are carrying the anti-mission have melted together with unity. Hence, in our sphere we still do not have the competence to process the shadow out of the world, but this is the goal that we are pursuing.

## THE FOUR EXISTENTIAL POSITIONS

Because the good and the evil are balancing inside us, before we have become conscious and have developed personally, we are absolutely free to choose our existential position. We have identified four such positions (see Fig. 2). The first position is indifferent in respect to our purpose of life. The second position is on the side of the purpose. The third is on the anti-purpose side. The fourth is a balanced position, which considers both the good and the evil, and integrates both, which is the fruitful path of personal development.

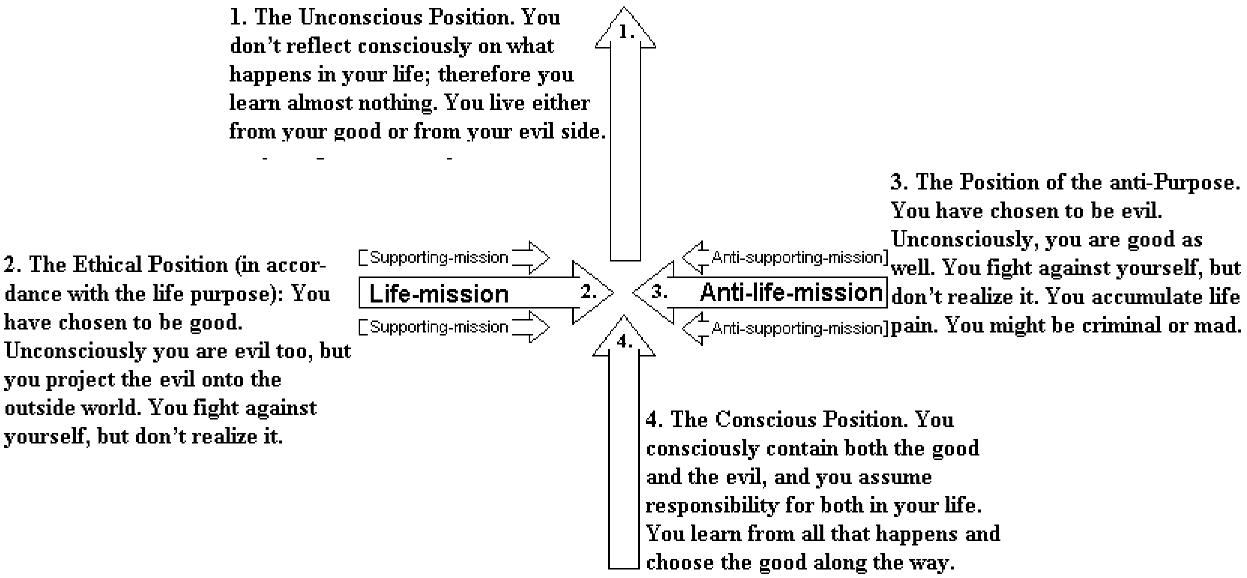

**FIGURE 2**. The human being can be unconscious and opportunistic. It can be half-conscious and choose either to be good or bad, or it can be conscious and admit both the good and the evil in itself, attempt to be good in spite of everything, and learn from anything that happens.

## 1. The Unconscious, Opportunistic Position

This position is the most common. It is not associated with the integration of the shadow and consequently gives no personal development. We live the purposes that occur to us, but there is no connection with the (personal) purpose of life. Therefore, the position is neither along with nor against the life purpose, but is regulated by the sum of the good and the evil in the person itself. So the whole lives its own life without any particular vigor, and the fragments carrying both the good and the evil intentions are active at a low stage and result in both good and evil things that are outside control of the person. Typically, this person will, without taking notice of it, alter between the positions good, evil, and indifferent, depending on what best serves survival and needs fulfillment. The opportunist position means that the person cannot be trusted or counted on. He or she is subject to outer circumstances, without observing his or her own intentions or the behavior resulting from these intentions. When that person wakes up and realizes that he or she has to choose between the good and the evil, he or she enters into one of the positions of the half-conscious human being[23].

## 2. The Half-Conscious, Ethical (or Good) Position

This position is not unusual. The person knows itself well enough to be aware of what life is and places itself on the side of the life. The difficulty of boosting its own life purpose without assuming the responsibility for the shadow is that the shadow is projected outwards so that others become evil, destructive, unsympathetic, or unkind. Consequently, this position gives a polarization that pushes the evil out to the world so that the partner, the colleague, or the children are now the negative ones, whereas the person itself is perpetually positive. Interestingly enough, it seems that the account of this position is not particularly positive as the shadow is somehow invited inside in a projective manner, and it is very hard for this person to get any love affair, working relations, and other relations to function.

Typically, this person is accused of being dominating, manipulating, or egocentric. There is much more development in the good position than in the unconscious one, where the perpetual striving for the good fails again and again due to the inevitably accompanying shadow, which on the whole spoils any aims. However, this gives dynamics and movement, which at last leads to the person taking responsibility for the shadow, resulting in the conscious transcendent position (described below). People who choose the good are in danger of having to fight with their own shadow side (sort of shadow boxing), and sometimes might justify their malice from a consideration of "to return evil for evil". The shift to the half-conscious, evil position often takes place without the person actually noticing it. From this point one might return to the resigned, unconscious starting position[24].

## 3. The Half-Conscious, Evil Position

This position is rare and it has terrible consequences. It is the position of the evil father, evil mother, Satanist or deliberate criminal. In its extreme, it is the position of the rapist, the child molester, the murderer, Hannibal the Cannibal, and Count Dracula. When we perceive our true nature as our anti-purpose and choose to comply with it, we become completely evil persons. The good is projected onto the outside world and becomes the issue to be fought or spoiled. If we do not know ourselves extremely well, i.e., both our good and evil sides, it can be very difficult to understand the person who chooses the evil. However, the evil has many existential advantages. To begin with, we intended the anti-purpose in order to survive and get out of excruciating life pain, and actually we survived, so as a start the evil has allowed us to keep life. Later on in life, choosing the evil means that we do not suffer as when we are choosing our good, true life purpose, which is so infinitely hard to carry into effect. In fact, the person proves a delight by choosing the evil corresponding to the pain by choosing the good. So choosing the evil is rewarded by delight as in the same way choosing the good is punished by existential pain.

Obviously, this is only at the beginning as long as we are not whole. When we heal, we realize that the pain that prevented us from carrying out the life purpose will be balanced by the joy of living. The pleasure of revenge is then caught up by a dreadful sorrow about hurting others, because this is in fact the opposite of what we really want, since we want to make ourselves useful to the world. Our life purpose is always good. Choosing the evil is not a solution at sight, but can be experienced as joyful and releasing at the very moment where we break through to the lowest existential layer. The difficulty of choosing the evil is that we create even more pain for ourselves than we already had. This makes it still more difficult to awaken to a conscious position. Usually, people sink to a resigned, unconscious position at a lower existential stage than before choosing the evil. However, it is possible to help this person along to reach the conscious, transcendent position provided the person itself wants to be helped to understand what the whole life scene is about. A positive attitude and unconditional love can sometimes help the bad person toward the position of the conscious human being[25].

## 4. The Conscious Learning Position

When we clearly see that we are basically good, but unable to live our life purpose due to an awful lot of repressed life pain, we have to assume the transcendent position. We are not able to be good, we are not able to be bad. We are not able to be, but we can be conscious and learn. Right now we are not able to know ourselves. We cannot understand ourselves, but we are able to express ourselves and be alert (awake). We live consciously, but we still have no control of life. We can do our utmost knowing that at this stage it is not good enough. The consciousness is painful and sweet at the same time. Meaningfulness is the finest sweetness, but being conscious of the powerlessness is painful. The understanding that the way forward is the way back to life, through confrontation of the historic sufferings, is animating. This is possible as an adult, but not as a child. The conscious position implies that the old life pains break out to the surface and are gradually admitted. When the comprehension is clear, a chaotic historic life scene is crystallized into one single or a few negative resolutions that can be let off now. So this life is in onward motion, is cleared, and the true life is returning slowly, without any inhibition, without any inner contrast or reluctance. So the consciously transcendent position, essentially to certify our own life, is a deep reflection of our soul, regardless of how painful it is to see the truth. This is what makes life evolve optimally[26].

One of the most difficult questions is why so few people reach the conscious learning position. The main obstacles seem to fall into two categories: inner and outer. The inner obstacle seems to be existential pain, as discussed above. The outer obstacle seems to be the need for social acceptance. A person who is learning must be honest with himself, which can be hard sometimes in an environment where other people are “playing games” and not willing to make a learning effort. This means that a person in the process of learning about him- or herself is also a source for learning in his or her environment. But learning goes together with existential pain, so this person actually often involuntarily inflicts pain on the people near and dear to him. So it seems that people in the conscious, learning position need people of their own kind to socialize with. This is perhaps the most severe obstacle facing the patient entering holistic existential therapy. You are entering a world of learning, of joys, meaning, and suffering, where most people do not go. So you will have to face loneliness and you will have to seek new friends. It is important for the holistic physician or therapist to build networks of patients and clients in personal growth, to avoid the feeling of being completely alone, and to avoid letting the patient be drawn into milieus and religious sects. This would not help the person in growth and may possibly also hinder the person’s self-actualization in the long run.

It is also very important to understand that the loneliness experienced when entering the learning process is often overwhelming, tempting the patient to choose position three (the half-conscious, evil position) instead, or even in the extreme — tempting the patient to consider suicide. Often the suicidal patient is caught between the positions two and four, two or three, or three and four. If the holistic physician can help the patient to understand the situation from an existential and developmental perspective, the patient will realize that entering position four is to prefer suicide, even if this position is emotionally very difficult. To die is really not the problem; the problem is to live, and to live a full and conscious life. As the Russian saying goes: “Death is easy, life is difficult”.

## RELEVANCE OF THE EXISTENTIAL THERAPY

The classical Greek virtues of beauty, goodness, and truth reflect three levels of existence, which Kierkegaard called the esthetical, the ethical, and the religious layers in man[27]. Søren Aabye Kierkegaard (1813–1855) was a prolific writer in the period called the "golden age" in Denmark of intellectual and artistic activity with work in the fields of philosophy, theology, psychology, literary criticism, and fiction. He was a social critic and wanted to renew Christian faith within Christendom. He is known as the "father of existentialism", but also for his critiques of Hegel and the German romantics, his contributions to the development of modernism, his literary experimentation, and talents to analyze and revitalize Christian faith. He burned with the passion of a religious poet, was armed with extraordinary dialectical talent, and drew on vast resources of erudition.

In the integrative quality of life theory[28], the dimension of beauty is reflected by man’s superficial layer connected with well being and satisfaction, needs fulfillment, and ability to function. The ethical layer deals with life expansion, whereas the religious layer concerns the meaning of life and a deep inner balance.

Through the theory of the anti-self, the ethical layer becomes accessible for existential therapy. The good and the evil manifest themselves in the patient as well as in the consciousness of the therapist and the patient is temporarily able to enter into the evil valence in order to confront it, mirrored from the outside by the therapist, and from the inside by its own good side. Through this double reflection, the light of the consciousness is thrown onto the patient's dark side, which brings the historic conglomerate of negative experiences and decisions amounting to man's dark side, the anti-self, or shadow, to collapse in a series of painful memory pictures, which can be belabored subsequently and integrated one at the time, e.g., by group therapy. By this process, the patient goes from the surface, the esthetical, first to the ethical layer and then, simultaneously with the inner contrasts being admitted and transcended, onto the deepest existential layer, which Kierkegaard called the religious[27].

## DISCUSSION

In connection with severe illness, it is often relevant to look at the unconscious evil. For instance, when one partner in a relationship is severely ill and the other is completely healthy, fit, extremely good, and considerate, this couple may have an unconscious agreement that one of them carries the darkness for them both, whereas the other partner carries the light. The bright, healthy party often appears full of energy, most considerate, and thoroughly devoted. At a closer analysis, the good party will at first appear dominating and later actually condemning and dissociating towards to the "evil" and ill party. Additionally, it often turns out that the apparently good person does not in fact have good intentions or love towards the ill partner. The bad side has been projected forth and over to the partner, who is now the intolerable and negative partner. The ill partner is not seen and met, and feels typically lonesome and unloved in spite of the healthy partner's apparent goodness and helpfulness. This loneliness and lack of mental meeting and understanding is typical of a relationship that is dominated by shadow projections. If the ill partner becomes healthy, it is necessary that both of them again assume the responsibility for both the bright and the dark sides. The process helping the patient to become healthy results preliminarily in the healthy partner getting more "ill". The responsibility for the dark side is being divided, and the poor fellow who carried all their common darkness can now be helped to confront and integrate its own darkness[29].

When the life purpose is clearly admitted and the negative decisions that deny it are found and let go of, the disorder often heals up even when this should not occur according to statistics. By means of the existential therapy which manages the shadow and brings the human being to its deepest existential layer, it thus seems possible to induce the spiritual arousal, which is so typical indeed in connection with the “spontaneous remissions” of, as an example, cancer[30].

Confronting and integrating the human being’s dark side make it also possible for people who are persuaded to be evil to change into seeing themselves as basically good. This change implies that their evil side has no longer free occasion to expand. The justification of the negative living, by describing the human nature as basically evil, is thus dissolved and no longer possible. The noble art of life called no-mind in the eastern traditions seems to be about suspending reason but to remain good. The famous and rare state of enlightenment seems to follow total integration of the shadow.

## CONCLUSIONS

Everybody has a dark side, an evil shadow that mirrors all the beautiful and lovely aspects of their soul. Knowing this and taking responsibility for both good and evil allows the person to take the learning position and little by little develop their consciousness and transcend their "shadow". In doing this, they integrate all their inner conflicts and let go of all their self-destructive decisions and attitudes, and so they become beautiful, good, and true.

Scientifically speaking, the "shadow" can only be examined by qualitative methods, where you apply your own consciousness to explore the consciousness of yourself and others. As it seems infinitely much easier to examine the shadow of the other person, friend, partner, client, or patient, everybody who wants to learn about his or her shadow is obliged to listen to the other, to know and understand himself. A fine way to see if what is learned in general is true is to make models and theories of the shadow and test these theories against reality. If a model or theory makes therapy easier and more successful, this is a good indicator that the theory in some way or another is true. But qualitative science is as tricky as consciousness itself; in the end what makes us choose a theory and call it valid is our personal liking and ability to understand life, our self, and our patient better from it.

As everybody seemingly owns a dark side, which makes big problems for us if not realized and integrated, we suggest that the shadow should be a theme in every course of holistic treatment. We suppose that many of the hidden resources needed to be well again are actually bound by the patents unconscious struggle with the shadow. It might be that understanding and integrating the shadow, confronting the evil, walking awake into the darkness to win it over, is the straight way to light, joy, love, self-exploration, and in the end healing. Only the one who carries the light of consciousness and conquers the darkness of lies and unconsciousness will reach the state of transcendence described by Maslow, or the state of coherence described by Antonovsky, or the state of meaning described by Frankl[6,9,16,17]. People who engage fully in the battle against the darkness often come to experience the whole universe as basically made of light. This light is often ascribed as brilliantly white and divine, or even as a unity, or a person, making it possible “to meet with G-d himself”.

As this last perspective is what motivates us in our work and our lives, and what urges us to develop our precious medical science in the service of mankind, we would like, in the end of these five papers on existential theory, to devote our final lines wholeheartedly to the divine being, or G-d. “These words which I command you today shall be *on* your heart” (Deut. 6:6). This statement to keep “these words” or G-d’s commandments above our heart, means that they supersede our own wishes and if you follow that path, it should be the beginning of the redemption of mankind from bestiality and the breaking down of his egotism, the root of evil in this world[3]. Rabbi Eleazar Ha-Kappar used to say[31]: “Those who are born are destined to die; those who are dead are destined to be brought to life again, and the living are destined to be judged. It is for you to know, proclaim and be sure that he is G-d. He is the Maker, he is the Creator, he is the Discerner, he is the Judge, he the Witness, he the Complainant and it is he who will judge. Blessed be he in whose presence there is no wrongdoing, not forgetting, nor partiality, nor taking bribes. Know that all is according to reckoning and let not your imagination persuade you that the grave is a place of refuge for you. Perforce you were formed and perforce you were born. Perforce you live, perforce you shall die and perforce you shall have to give a strict account before the Supreme King of kings, the Holy One, blessed be He”.

So whoever believes and accepts the yoke of heaven will understand the transience and unimportance of this world, as well as the eternity and infinity of the world to come, where the soul will find its permanent place. This person will also understand that the eradication of evil from this world is only a hairsbreadth of difference between his dying now and dying naturally according to his normal life span[32]. Rabbi Jacob made a more simple conclusion[33]: “This world is like a vestibule before the world to come. Prepare yourself that you may enter into the banquet hall”.

## ACKNOWLEDGMENTS

This study was supported by grants from The 1991 Pharmacy Foundation, as well as by supplementary grants from Goodwill-fonden, the JL-Foundation, E. Danielsen and Wife's Foundation, Emmerick Meyer's Trust, the Frimodt-Heineken Foundation, the Hede Nielsen Family Foundation, Petrus Andersens Fond, Wholesaler C.P. Frederiksens Study Trust, Else & Mogens Wedell-Wedellsborg's Foundation and IMK Almene Fond. The research was approved by the Copenhagen Scientific Ethical Committee under number (KF)V.100.2123/91.

## BIOSKETCHES

**Søren Ventegodt, MD,** is the Director of the Quality of Life Research Center in Copenhagen, Denmark. He is also responsible for a Research Clinic for Holistic Medicine in Copenhagen and is a popular speaker throughout Scandinavia. He has published numerous scientific or popular articles and a number of books on holistic medicine, quality of life, and quality of working life. His most important scientific contributions are the comprehensive SEQOL questionnaire, the very short QoL5 questionnaire, the integrated QOL theory, the holistic process theory, the life mission theory, and the Danish Quality of Life Research Survey, 1991–94 in cooperation with the University Hospital of Copenhagen and the late pediatric professor Bengt Zachau-Christiansen. E-mail: ventegodt@livskvalitet.org. Website: www.livskvalitet.org/

**Niels Jørgen Andersen, MSc,** Professor, Department of Innovation and Economic Organization, Norwegian School of Management. This department conducts research and provides teaching in central topics related to innovation, business development, management of global companies, business history, and economic organization. Research activities within the Department are related to four core subjects within the discipline: business history, cooperative organizations, business development and entrepreneurship, and finally studies of industries with a special focus on the electricity industry. He is also the dynamic chairman of the nonprofit organization Stiftelsen Holistisk Medisin Scandinavia, which aims to support the scientific development, research, and documentation of complementary and holistic medicine in Scandinavia. E-mail: niels.j.andersen@bi.no. Website: www.bi.no/users/fgl93013/

**Joav Merrick, MD, DMSc,** is Professor of Child Health and Human Development affiliated with the Zusman Child Development Center, Division of Pediatrics and Community Health at the Ben Gurion University, Beer-Sheva, Israel; the Medical Director of the Division for Mental Retardation, Ministry of Social Affairs, Jerusalem; and the Founder and Director of the National Institute of Child Health and Human Development. He has numerous publications in the field of child and human development, rehabilitation, intellectual disability, disability, health, welfare, abuse, advocacy and prevention. Dr. Merrick received the Peter Sabroe Child Award for outstanding work on behalf of Danish Children in 1985 and the International LEGO-Prize ("The Children's Nobel Prize") for an extraordinary contribution towards improvement in child welfare and well being in 1987. E-mail: jmerrick@internet-zahav.net. Website: www.nichd-israel.com
